# Circulating Vitamin D and Risk of Epithelial Ovarian Cancer

**DOI:** 10.1155/2009/672492

**Published:** 2009-08-27

**Authors:** Alan A. Arslan, Tess V. Clendenen, Karen L. Koenig, Johan Hultdin, Kerstin Enquist, Åsa Ågren, Annekatrin Lukanova, Hubert Sjodin, Anne Zeleniuch-Jacquotte, Roy E. Shore, Göran Hallmans, Paolo Toniolo, Eva Lundin

**Affiliations:** ^1^Department of Environmental Medicine, New York University School of Medicine, 550 First Avenue, New York, NY 10016, USA; ^2^Department of Obstetrics and Gynecology, New York University School of Medicine, 550 First Avenue, New York, NY 10016, USA; ^3^New York University Cancer Institute, New York, NY 10016, USA; ^4^Department of Medical Biosciences, University of Umeå, 901 87 Umeå, Sweden; ^5^Department of Public Health and Clinical Medicine/Nutritional Research, University of Umeå, 901 87 Umeå, Sweden; ^6^Division of Cancer Epidemiology, German Cancer Research Center (DKFZ), 69120 Heidelberg, Germany; ^7^Radiation Effects Research Foundation, Hiroshima 732-0815, Japan

## Abstract

We conducted a nested case-control study within two prospective cohorts, the New York University Women's Health Study and the Northern Sweden Health and Disease Study, to examine the association between prediagnostic circulating levels of 25-hydroxy vitamin D (25(OH)D) and the risk of subsequent invasive epithelial ovarian cancer (EOC). The 25(OH)D levels were measured in serum or plasma from 170 incident cases of EOC and 373 matched controls. Overall, circulating 25(OH)D levels were not associated with the risk of EOC in combined cohort analysis: adjusted OR for the top tertile versus the reference tertile, 1.09 (95% CI, 0.59–2.01). In addition, there was no evidence of an interaction effect between *VDR* SNP genotype or haplotype and circulating 25(OH)D levels in relation to ovarian cancer risk, although more complex gene-environment interactions may exist.

## 1. Introduction

There is considerable interest in understanding the role of vitamin D in cancer in general and in ovarian cancer in particular. Experimental studies have shown that vitamin D administration reduces proliferation and promotes apoptosis in ovarian cancer cell lines and animal models [1–6]. Ovarian cancer incidence and mortality rates are higher in northern latitudes, where sun exposure, which is required for the initiation of vitamin D synthesis in the skin, is lower [7–10]. An inverse association between dietary vitamin D intake and risk of ovarian cancer has been reported in one epidemiologic study [11], although others have not supported this finding [12–16]. Studies of the effect of dietary vitamin D intake are limited because estimation of dietary vitamin D intake does not capture cutaneous production of vitamin D. Thus, circulating vitamin D, which reflects both cutaneous production and dietary/supplement intake [17–20], is considered the best indicator of overall vitamin D status.

1,25(OH)_2_D is the biologically active vitamin D metabolite. However, its concentration is about 1000 times lower than that of 25(OH)D in circulation, due to its shorter half-life and local production in target tissues, such as the ovaries [21, 22]. Thus, 25(OH)D is thought to better reflect overall vitamin D status than 1,25(OH)_2_ D [23, 24]. 

To date, only one epidemiologic study has examined the relationship between prediagnostic levels of vitamin D and risk of ovarian cancer. Tworoger et al. did not find an association of ovarian cancer risk with the 25(OH)D or 1,25-dihydroxyvitamin D (1,25(OH)_2_D) plasma levels [25].

Polymorphisms in the *VDR* gene may influence the ability of 1,25(OH)_2_D to activate vitamin D target genes, including those involved in growth regulation and apoptosis. In a previous manuscript, we reported an overall null association between four common polymorphisms in the *VDR* gene (*Bsm1*, *Apa1*, *Taq1*, and *Fok1*) and risk of ovarian cancer [26], although a retrospective multiethnic study of 313 cases and 574 controls [27] and a large pooled analysis including 1473 cases and 2006 controls from four studies (one retrospective case-control study and three case-control studies nested in prospective cohorts) [28] found an association with the *Fok1* SNP among Caucasian women. Mixed results for this and other *VDR* SNPs may be due to chance findings or limited power to detect associations [27]. It is also possible that genetic variants influence ovarian cancer risk differently, depending on an individual's vitamin D status. 

The objective of the present study was to examine the relationship between circulating levels of 25(OH)D and risk of invasive epithelial ovarian cancer and to assess the combined effect of circulating 25(OH)D and *VDR* polymorphisms on ovarian cancer risk.

## 2. Methods

### 2.1. Study Population

Descriptions of the University of Umeå Northern Sweden Health and Disease Study (NSHDS) and the New York University Women's Health Study (NYUWHS) have been provided previously [29–31]. Briefly, since 1985 the NSHDS has enrolled approximately 53 756 women aged 30–65 through local health promotion intervention programs in Northern Sweden. The NYUWHS enrolled 14 274 healthy women aged 34–65 years at a breast cancer screening center in New York City between 1985 and 1991. Each study cohort collected information about medical history, reproductive history, family history of cancer, medications, smoking history, and diet during enrollment and/or followup. Blood collected at enrollment and any subsequent visits was processed according to standardized procedures within each cohort. In the NSHDS cohort, blood was collected, centrifuged, and plasma aliquots were frozen at −80°C and transferred within 1 week to a −80°C central storage facility (the Northern Sweden Medical Research Biobank). In the NYUWHS cohort, blood was drawn, collection tubes were kept covered at room temperature (21–25°C) for 15 minutes, then at 4°C for 60 minutes to allow clot retraction, and then centrifuged for 25 minutes. After centrifugation, serum samples were divided into aliquots and immediately stored at −80°C at the local site. Participants who reported being pregnant or using exogenous hormones within 6 months of enrollment were not eligible for the NYUWHS cohort or for case-control selection from NSHDS.

### 2.2. Case Ascertainment

In the NSHDS, cohort linkages to regional and national cancer registries and to all-cause mortality registries were used to capture cases of incident invasive epithelial ovarian cancer. In the NYUWHS cohort, case ascertainment was achieved through self report on followup questionnaires and through linkages with state tumor registries in New York, New Jersey, and Florida. Medical records were obtained to verify reported events. As of November 1, 2005, a total of 192 invasive ovarian cancer cases (107 from the NSHDS and 85 from the NYUWHS) had been identified. Twenty-two nonepithelial ovarian cancer cases (8 from the NSHDS and 14 from the NYUWHS) were excluded from this study because they did not meet the criteria of being invasive epithelial ovarian cancer. For the current study, there were a total of 170 invasive EOC cases (71 from NYUWHS and 99 from NSHDS). However, two cases from the NSHDS cohort had only DNA available for analysis, and were only included in the main *VDR* genotyping analysis [26].

### 2.3. Selection of Controls

For each case, two controls were randomly selected from cohort members who were alive and free of cancer at the time of diagnosis of the case. Controls were also matched to the case on cohort (NYUWHS or NSHDS), age at entry (±6 months) and date of blood donation (±15 days). Seventeen cases from the NYUWHS cohort had only one eligible matched control because 10 controls had a complete bilateral oophorectomy before the diagnosis date of the case and seven cases could not be sufficiently closely matched to a second control on date of blood donation (strict matching is required to control for seasonal effects of vitamin D). Of the potential 323 eligible controls in total (198 from the NSHDS, 125 from the NYUWHS), seven were excluded from the NSDHS because either the control or matching case did not have plasma available.

The institutional review boards of New York University School of Medicine and the Regional Ethical Committee of the University of Umeå, Sweden, and the Swedish Data Inspection Board reviewed and approved this study.

### 2.4. Laboratory Methods

Serum 25(OH)D was measured at the University of Umeå using a gamma-B 25-hydroxy vitamin D radioimmunoassay (Immunodiagnostic Systems (IDS), Inc.). Acetonitrile extraction of 25(OH)D was followed by incubation with radioionidated tracers and sheep antibodies to 25(OH)D. Antibody-bound tracer is inversely proportional to the concentration of 25(OH)D. Samples from each cohort and case-control set were assayed together in the same laboratory batch. Quality controls and study samples were distributed randomly throughout the batches and the laboratory personnel were blinded to the samples' case-control status. The intra- and interbatch coefficients of variation were 17.7% and 24.6% for NSHDS, respectively. For NYUWHS, the intra- and interbatch coefficients of variation were 16.6% and 16.2%, respectively. 

Genotyping was performed at New York University School of Medicine. Methods, quality control procedures, and genotyping success rates have been reported previously [26].

### 2.5. Statistical Analysis

25(OH)D values were log _2_ transformed to reduce departures from the normal distribution (the log _2_ transformation is useful for estimating odds ratios associated with a doubling in vitamin D levels)**.** Two control participants in the NYUWHS cohort with outlying values for 25(OH)D (>150 nmol/L) were set to missing. Odds ratios from analyses including or excluding these participants did not differ appreciably. Linear regression and ANOVA were used to test whether 25(OH)D levels differed according to baseline characteristics. Conditional logistic regression, which allows for the matched design, was used to evaluate the association between serum concentration of 25(OH)D and risk of EOC. Because cases were individually matched to controls for laboratory batch and date of sampling (i.e., season), these variables were controlled for by design when 25(OH)D was modeled as a continuous variable. To create meaningful tertiles of 25(OH)D for categorical analysis, however, the 25(OH)D values needed to be laboratory batch and season, to prevent individuals from being arbitrarily included in a tertile simply on the basis of their season of blood draw or inclusion in a particular batch. The adjustment for laboratory batch was done by first regressing the log-transformed 25(OH)D values on season, age, and BMI (the three variables that were associated with 25(OH)D) and computing the residuals within each cohort. We then computed the mean residual for each laboratory batch and subtracted the appropriate batch mean from each individual's log-transformed vitamin D value to center each batch at the grand mean for each cohort. We then adjusted for seasonal effects by performing a nonparametric local regression (Proc Loess, SAS) of 25(OH)D values on day of the year of blood donation and used the residuals to create cohort-specific tertiles [32]. Potential confounders, specifically reproductive history (parity, number of full-term pregnancies, and age at first full-term pregnancy), age at menarche, menopausal status at enrollment, history of oral contraceptive use at baseline, body mass index at enrollment, and smoking status at baseline (never, current, former) were considered in the logistic regression models. However, the only variables significantly different between cases and controls were parity and oral contraceptive use (both categorized as ever/never). Therefore, multivariate models included only these two variables (age and date of blood donation which were controlled for by the matched design and use of the conditional logistic regression model). An indicator variable for sample type (serum versus plasma) also was examined as a potential interaction term. We also conducted analyses excluding individuals diagnosed within five years of blood donation, and evaluated the vitamin D-ovarian cancer relationship within histological subtypes, and by stage (I-II versus III-IV), grade, BMI (dichotomous variable < 25 versus ≥25), oral contraceptive use (ever versus never), and *VDR* SNP genotype (assuming a codominant model). Haplotypes were estimated from genotype data using PHASE version 2.1.1 (http://www.stat.washington.edu/stephens/phase.html) and odds ratios for the interaction between having zero, one, or two copies of the haplotype and 25(OH)D levels were determined using conditional logistic regression. We used SAS software (version 9.1, SAS institute, Cary, NC) for all statistical analyses.

## 3. Results

The characteristics of the study participants from each cohort at baseline have been described previously [26]. An abbreviated description is shown in Table 1. The median age at enrollment was 52 and 55 years for NSHDS and NYUWHS participants, respectively. NSHDS cases had a median of 4.6 years between blood donation and diagnosis and NYUWHS cases were diagnosed an average of 7.0 years after blood donation. NSHDS cases were less likely than NSHDS controls to have ever been pregnant (78% versus 89%, *P* = .01) and to have ever taken oral contraceptives (33% versus 45%, *P* = .03). NYUWHS cases and controls were not significantly different with regard to their baseline characteristics. Regarding differences between cohorts, NYUWHS controls were more likely than NSHDS controls to be nulliparous (31% versus 11%, *P* < .0001), more likely to be premenopausal at baseline (42% versus 28%, *P* = .01), less likely to have used oral contraceptives (32% versus 45%, *P* = .03), more likely to have smoked (61% versus 43%, *P* = .01), and had higher 25(OH)D levels (45.8 versus 39.4 nmol/L, *P* = .04). Cases and controls did not differ with regard to genotype frequency at any of the four *VDR* SNP sites for either of the cohorts [26].

Table 2 shows 25(OH)D by characteristics of controls for the NSHDS and NYUWHS cohorts. In both cohorts, median 25(OH)D levels were higher in women who were older at enrollment and in women who had lower BMI, and were somewhat higher in women who were ever parous and had used oral contraceptives. However, the two cohorts differed with regard to smoking status: in the NYUWHS, current smokers had higher median 25(OH)D levels (50 nmol/L in current versus ~45 nmol/L in former and never smokers), while smoking in the NSHDS showed a somewhat opposite relationship with 25(OH)D (~38 nmol/L in current and former versus 40 nmol/L in never smokers). 25(OH)D was only modestly related to *VDR* genotype in the NSHDS cohort at the *Bsm1* (*P* = .16) and *Taq1* (*P* = .19) loci (Table 3). In both cohorts, women with two copies of the baT haplotype (associated with the *Bsm1* G, *Apa1* G, and *Taq1* T allele combination) had higher levels of circulating 25(OH)D, although the tests for trend were not statistically significant (Table 3).

We did not observe an overall association between vitamin D and ovarian cancer when 25(OH)D was modeled on the continuous (multivariate adjusted OR for a doubling in vitamin D levels = 1.1, 95% CI: 0.7–1.7 ) or categorical scale (Table 4). There was some evidence of interaction between 25(OH)D and cohort (*P* = .07), though cohort-specific odds ratios do not support an association with risk in either cohort (Table 4). The lack of association between vitamin D and ovarian cancer remained after controlling for parity and oral contraceptive use. We did not observe any significant differences in the relationship between 25(OH)D and ovarian cancer by season of blood collection, BMI (<25 versus ≥25 kg/m^2^), menopausal status at enrollment, *VDR* SNP genotype or haplotype, tumor stage (I-II versus III-IV), grade, or histological subtype (serous versus mucinous, clear cell, and endometriod) (data not shown). Odds ratios for 25(OH)D and risk of ovarian cancer did not change appreciably in analyses restricted to 74 matched sets in which cases were diagnosed five or more years after blood donation (OR for the highest versus lowest tertile = 0.9, 95% CI: 0.46–1.72).

## 4. Discussion

In the current study, we did not observe an association between circulating levels of 25(OH)D and risk of epithelial ovarian cancer. The lack of an association remained after controlling for potential confounders, excluding cases diagnosed within 5 years of blood donation, and considering subgroups defined by genetic variation in the *VDR*, tumor characteristics (i.e., stage, grade, and histological subtype), menopausal status, and BMI.

Initial support for a protective role for vitamin D was provided by ecological studies showing an inverse association between UV-B exposure and ovarian cancer incidence or mortality rates [7–10]. However, research conducted with this type of study design cannot elucidate the temporal association between exposure and disease. Case-control and cohort studies of dietary vitamin D intake and risk of ovarian cancer have been inconsistent [11–16]. The results of our study are in agreement with the overall results of Tworoger et al. [25], the only other prospective study of ovarian cancer to report on circulating 25(OH)D to date. Our study similarly found that the lack of association between serum 25(OH)D and risk of ovarian cancer did not differ by *VDR* genotype [28]. However, Tworoger et al. found an inverse association for 25(OH)D among women with BMI ≥ 25 kg/m^2^ [25], which was not observed in the current study.

Consistent with previous studies [33–37], higher BMI was associated with lower circulating 25(OH)D in both cohorts. Vitamin D is fat-soluble and studies in obese persons and mice have shown increased storage and decreased bioavailability of vitamin D in fat cells [38, 39]. We also observed that parity was associated with higher 25(OH)D; which is in agreement with a similar observation in the PLCO Cancer Screening Trial [37]. Contrary to some previous reports [37, 40, 41], we observed a positive relationship between age and 25(OH)D, which showed increased levels of vitamin D in the 50–60 year age group as compared with younger women. This may be due to an increased intake of calcium and vitamin D-rich foods and supplements around the time of menopause in an effort to prevent or treat osteoporosis. Oral contraceptive use was associated with higher median 25(OH)D levels in our study; previous studies have found a similar relationship in current OC users [17, 42, 43]. We also found that the *Bsm1* G, *Apa1* G, and *Taq1* T haplotype, in the *VDR* gene was modestly associated with higher 25(OH)D. Publications on the relationship between *VDR* genotype and levels of 25(OH)D have been fairly inconsistent. In the Physicians Health Study Cohort, the *Bsm1* genotype was not related to total 25(OH)D levels, although the BB genotype was associated with higher index of free 25(OH)D and with higher 1,25(OH)_2_D levels in older men [44]. The BB genotype was also associated with higher 25(OH)D in a small Finnish study (*n* = 93) that collected all blood samples in the winter when skin synthesis of vitamin D is very low [45]. However, a large study of 2845 women found no association between any *VDR* SNPs genotype and 25(OH)D [46]. Individuals with two copies of the bAT haplotype (*Bsm1* G, *Apa1* T, and *Taq1* T) had lower levels of 25(OH)D than individuals with 0 or 1 copy (*P*-trend = .09) [46]. Individuals without any copies of baT (*Bsm1* G, *Apa1* G, and *Taq1* T) in the current study (which would include all individuals with two copies of the bAT haplotype) had lower 25(OH)D levels, thus providing indirect support for the previous finding. 

This study limitations include the use of only one sample to determine each participant's 25(OH)D status. However, in a preliminary reproducibility study of 25(OH)D among 16 NYUWHS participants with at least 3 annual visits, the intraclass correlation coefficient was quite high (0.71), suggesting stability of this metabolite from visit to visit, which has been observed by others [47, 48]. The relatively high coefficient of variation for the 25(OH)D assay adds random error to the measurements and may have attenuated the association. Potential residual confounding and relatively small sample size are additional limitations. Although we had sufficient sample size to detect small differences between cases and controls (a priori, we had 80% power to detect a mean difference of 6 nmol/L between cases and controls), we had limited ability to explore interactions. Previous studies have demonstrated that vitamin D deficiency is more common in Europe than the United States, and that in the United States, the latitude of residence is inversely associated with vitamin D (reviewed in [49]). We anticipated that the ranges of 25(OH)D values in the New York and Northern Sweden populations would be lower than those reported from lower latitudes [50, 51]. In the highest tertile, the median value for 25(OH)D was 68.9 nmol/L for NYUWHS and 51.6 nmol/L for NSHDS; somewhat lower than the 75 nmol/L, that is, considered to be optimal for multiple health outcomes [52]. Few women in our study had levels above 75 nmol/L, thus we cannot rule out the possibility that women with very high 25(OH)D levels may have a reduced risk of ovarian cancer. Strengths of the study include the use of samples collected before diagnosis, limiting the potential for existing disease to influence vitamin D levels, the use of circulating 25(OH)D as a composite measure of vitamin D overall status, careful consideration of seasonal effects, and the use of a nested case-control design, which helps ensure that the controls are comparable to the cases. 

In conclusion, our findings do not provide support for the hypothesis that circulating vitamin D levels are associated with risk of invasive epithelial ovarian cancer later in life. Larger studies are needed to evaluate gene-environment interactions and potential subgroups which may benefit from vitamin D chemoprevention.

## Figures and Tables

**Table 1:** Characteristics of invasive epithelial ovarian cancer cases and matched controls, NYUWHS and NSHDS cohorts.

Characteristic	NYUWHS		NSHDS	
	Cases (*n* = 71)	Controls (*n* = 125)	Cases (*n* = 97)	Controls (*n* = 191)
Age at sampling, median (25%–75%), y	55 (47–62)	55 (46–62)	52 (50–60)	51 (50–60)
Time to diagnosis, *median (25%–75%), y	7.0 (3.7–9.5)	—	4.6 (2.0–7.1)	—
BMI at sampling, median (25%–75%), kg/m^2^	25 (22–28)	25 (23–28)	25 (23–28)	25 (23–28)

Menopausal status at enrollment, ^†^ *n* (%)				
Premenopausal	29 (40.8)	52 (41.6)	30 (30.9)	53 (27.7)
Postmenopausal	42 (59.2)	73 (58.4)	67 (69.1)	138 (72.3)

Parity, ^†,‡^ *n* (%)				
Never	28 (39.4)	39 (31.2)	18 (22.5)	19 (11.0)
Ever	43 (60.6)	86 (68.8)	62 (77.5)	153 (89.0)

OC use, ^†,‡^ *n* (%)				
Never	39 (70.9)	63 (68.5)	54 (67.5)	89 (54.6)
Ever	16 (29.1)	29 (31.5)	26 (32.5)	74 (45.4)
Unknown				

Smoking status, ^†^ *n* (%)				
Never	25 (40.3)	38 (38.8)	53 (61.6)	100 (56.8)
Ever	37 (59.7)	60 (61.2)	33 (38.4)	76 (43.2)

25(OH)D, median (25%–75%), nmol/L	47.8 (31.4–64.5)	45.8 (28.1–59.8)	37.6 (29.5–46.5)	39.4 (32.8–47.8)

*Differs significantly between NYUWHS and NSHDS cases at *P* = .0001. ^†^NYUWHS and NSHDS controls differ significantly (*P* < .05) with regard to age at menarche, menopausal status, parity, ever use of oral contraceptives, smoking status, and 25(OH)D. ^‡^Case-control differences significant only for NSHDS for parity (*P* = .01) and for oral contraceptives use (*P* = .03).

**Table 2:** Circulating 25(OH)D levels by baseline characteristics, NYUWHS and NSHDS controls.

Characteristic*	25(OH)D by Cohort (nmol/L)					
	NYUWHS			NSHDS		
	*N*	Median	(25%–75%)	*N*	Median	(25%–75%)
Age at sampling, y						
* *≤50 years	42	40.5	(24.6–64.0)	91	37.5	(24.1–53.8)
* *50–60 years	46	45.9	(30.7–66.7)	71	43.7	(28.4–68.1)
* * >60 years	35	44.9	(36.0–59.7)	29	39.4	(33.4–58.9)
*P*-trend (continuous)		.15			.007	

BMI at enrollment, kg/cm^2^						
* * <25	60	51.6	(31.3–67.2)	89	43.4	(29.7–65.8)
* *25–29	39	43.4	(29.7–53.8)	69	35.8	(24.1–50.2)
* *≥30	21	32.0	(25.5–44.6)	26	38.3	(24.8–61.0)
*P*-trend (continuous)		.004			.05	

Menopausal status at enrollment						
* *Premenopausal	51	37.5	(25.1–63.6)	53	34.8	(23.4–49.5)
* *Postmenopausal	72	45.6	(32.7–67.2)	138	42.2	(28.1–65.8)
*P*-value		.03			.002	

Parity						
* *Never	39	41.9	(29.7–65.8)	19	35.8	(21.9–53.4)
* *Ever	84	45.6	(29.4–64.0)	153	39.7	(25.6–61.8)
*P*-value		.49			.14	

Oral contraceptives use						
* *Never	62	44.6	(30.9–68.1)	89	38.6	(24.8–61.0)
* *Ever	28	49.5	(30.1–61.4)	74	42.5	(24.8–62.2)
*P*-value		.46			.67	

Smoking status						
* *Never	37	45.9	(30.7–61.4)	100	40.8	(26.7–64.0)
* *Former	39	44.6	(31.6–66.7)	40	38.1	(25.5–60.1)
* *Current	20	49.9	(27.9–66.3)	36	37.5	(22.0–54.9)
*P*-value		.62			.12	

Season						
* *Jan–Mar	29	38.1	(26.7–45.9)	63	34.1	(24.1–58.9)
* *Apr–Jun	26	42.5	(26.2–53.8)	44	40.8	(29.2–60.1)
* *Jul–Sep	40	49.9	(30.7–69.1)	16	46.2	(35.8–62.2)
* *Oct–Dec	28	55.7	(34.3–65.3)	68	43.4	(24.3–62.2)
*P*-value		.13			.02	

*All variables had missing data for fewer than 7 controls except for NHSDS: parity (19 missing), oral contraceptives use (28 missing), and smoking (15 missing), and for NYUWHS: oral contraceptives use (33 missing) and smoking (27 missing).

**Table 3:** Circulating 25(OH)D levels by vitamin D receptor Fok1 genotype and Bsm1, Apa1, and Taq1 haplotype, NYUWHS and NSHDS controls.

Characteristic	25(OH)D by Cohort (nmol/L)					
	NYUWHS			NSHDS		
	*N*	Median	(25%–75%)	*N*	Median	(25%–75%)
*Fok1*						
* *C/C	50	42.2	(25.5–68.6)	68	37.0	(24.3–53.8)
* *C/T	56	46.9	(30.5–64.0)	92	42.5	(24.8–65.8)
* *T/T	17	48.8	(31.1–64.4)	29	36.0	(26.2–62.2)
*P*-trend		.27			.28	

Haplotype 1						
(*Bsm*1 G, *Apa*1 G, and *Taq*1 T)						
* *0 copies	38	44.6	(32.0–53.8)	48	38.9	(24.1–60.5)
* *1 copies	60	44.6	(26.4–69.1)	93	38.6	(24.8–62.2)
* *2 copies	25	48.8	(30.7–63.1)	50	43.1	(26.4–62.2)
*P*-trend		.71			.25	

**Table 4:** Odds ratios for invasive epithelial ovarian cancer by tertile of 25(OH)D.

	ORs for season-adjusted 25(OH)D tertiles			*P*-trend
	Tertile 1	Tertile 2	Tertile 3	
NYUWHS	≤36.7 nmol/L	36.8–57.7 nmol/L	≥57.8 nmol/L	
*n*, case/control	22/42	23/43	26/38	
Model 1*	1.0 (reference)	0.97 (0.45–2.06)	1.35 (0.59–3.09)	0.50
Model 2^†^	1.0 (reference)	1.13 (0.39–3.27)	1.50 (0.53–4.23)	0.44

NSHDS	≤34.0 nmol/L	34.1–44.7 nmol/L	≥44.8 nmol/L	
*n*, case/control	37/58	28/70	32/63	
Model 1*	1.0 (reference)	0.62 (0.33–1.11)	0.79 (0.42–1.46)	0.49
Model 2^†^	1.0 (reference)	0.54 (0.25–1.17)	0.83 (0.38–1.81)	0.78

Combined cohorts	Cohort-specific cut points	Cohort-specific cut points	Cohort-specific cut points	
*n*, case/control	59/100	51/113	58/101	
Model 1*	1.0 (reference)	0.74 (0.46–1.20)	0.96 (0.59–1.58)	0.88
Model 2^†^	1.0 (reference)	0.78 (0.42–1.43)	1.09 (0.59–2.01)	0.71

*Conditional logistic regression model controlling for matching factors only: cohort, age at entry, and date of blood donation. ^† ^Conditional logistic regression model controlling for matching factors and additionally adjusted for oral contraceptive use (ever/never) and parity (ever/never) after exclusion of the participants with missing data for these variables (NYUWHS: *n* = 49, NSHDS: *n* = 49).
